# Dimethyl 5-acetyl-1-hy­droxy-4-methyl-1*H*-pyrrole-2,3-di­carboxyl­ate, an oxidation-resistant *N*-hy­droxy­pyrrole

**DOI:** 10.1107/S1600536813034466

**Published:** 2014-01-04

**Authors:** Gerald M. Rosen, Sukumaran Muralidharan, Peter Y. Zavalij, Joseph P. Y. Kao

**Affiliations:** a Department of Pharmaceutical Sciences, School of Pharmacy, University of Maryland, Baltimore, Maryland 21201, USA; b Center for Biomedical Engineering and Technology, School of Medicine, University of Maryland, Baltimore, MD 21201, USA; c Department of Chemistry and Biochemistry, University of Maryland, College Park, MD 20742, USA; d Department of Physiology, School of Medicine, University of Maryland, Baltimore, MD 21201, USA

## Abstract

The title compound, C_11_H_13_NO_6_, exhibits an intra­molecular O–H⋯O=C hydrogen bond between the *N*-hydroxyl H atom and carbonyl O atom of the neighboring acetyl group. This finding contradicts a previously published model in which the hydrogen bond was postulated to occur with the neighboring carbomethoxy group. This relatively strong hydrogen bond [O—H⋯O: *D* = 2.5583 (11) Å and θ = 152°] may underlie the resistance of the title compound to oxidation into the corresponding nitroxide.

## Related literature   

The title compound was obtained as part of an effort to synthesize aromatic nitroxides and was prepared by a published procedure (Hekmatshoar *et al.*, 2008 ▶). The compound could not be converted to the corresponding nitroxide under commonly used oxidation conditions (Keana *et al.*, 1988 ▶). For analysis of intra­molecular hydrogen-bond parameters in organic crystals, see: Bilton *et al.* (2000 ▶); Galek *et al.* (2010 ▶). A survey of the effect of intra­molecular hydrogen bonding on the reduction potential of quinones appears in the review by Guin *et al.* (2011 ▶). Examples of hydrogen bonding affecting the redox properties of quinones are discussed by Gupta & Linschitz (1997 ▶) and Feldman *et al.* (2007 ▶).
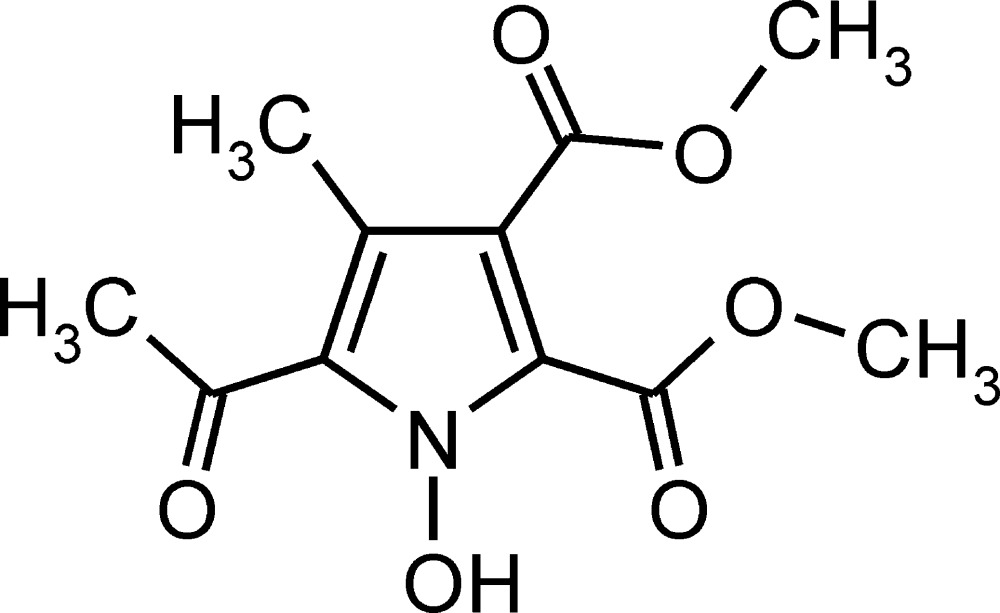


## Experimental   

### 

#### Crystal data  

C_11_H_13_NO_6_
*M* *_r_* = 255.22
Monoclinic, 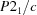

*a* = 10.3893 (8) Å
*b* = 15.1803 (12) Å
*c* = 7.5789 (6) Å
β = 99.630 (1)°
*V* = 1178.45 (16) Å^3^
*Z* = 4
Mo *K*α radiation
μ = 0.12 mm^−1^
*T* = 150 K
0.52 × 0.43 × 0.31 mm

#### Data collection  

Bruker SMART APEXII diffractometer
Absorption correction: multi-scan (*SADABS*; Sheldrick, 1996 ▶) *T*_min_ = 0.888, *T*_max_ = 0.964
19259 measured reflections
3437 independent reflections
2836 reflections with *I* > 2σ(*I*)
*R*_int_ = 0.014

#### Refinement  

*R*[*F* ^2^ > 2σ(*F* ^2^)] = 0.031
*wR*(*F* ^2^) = 0.064
*S* = 1.00
3437 reflections
215 parameters
All H-atom parameters refined
Δρ_max_ = 0.35 e Å^−3^
Δρ_min_ = −0.19 e Å^−3^

### 

Data collection: *APEX2* (Bruker, 2010 ▶); cell refinement: *SAINT* (Bruker, 2010 ▶); data reduction: *SAINT*; program(s) used to solve structure: *SHELXS97* (Sheldrick, 2008 ▶); program(s) used to refine structure: *SHELXL2012* (Sheldrick, 2008 ▶); molecular graphics: *XSHELL* (Bruker, 2010 ▶) and *Mercury* (Macrae *et al.*, 2008 ▶); software used to prepare material for publication: *publCIF* (Westrip, 2010 ▶).

## Supplementary Material

Crystal structure: contains datablock(s) I. DOI: 10.1107/S1600536813034466/ld2115sup1.cif

Structure factors: contains datablock(s) I. DOI: 10.1107/S1600536813034466/ld2115Isup2.hkl

Supporting information file. DOI: 10.1107/S1600536813034466/ld2115Isup3.cml

CCDC reference:

Additional supporting information:  crystallographic information; 3D view; checkCIF report

## Figures and Tables

**Table 1:** Hydrogen-bond geometry (Å, °)

*D*—H⋯*A*	*D*—H	H⋯*A*	*D*⋯*A*	*D*—H⋯*A*
O1—H1⋯O5	0.888 (16)	1.746 (16)	2.5583 (11)	150.8 (14)

**Fig. 1.**
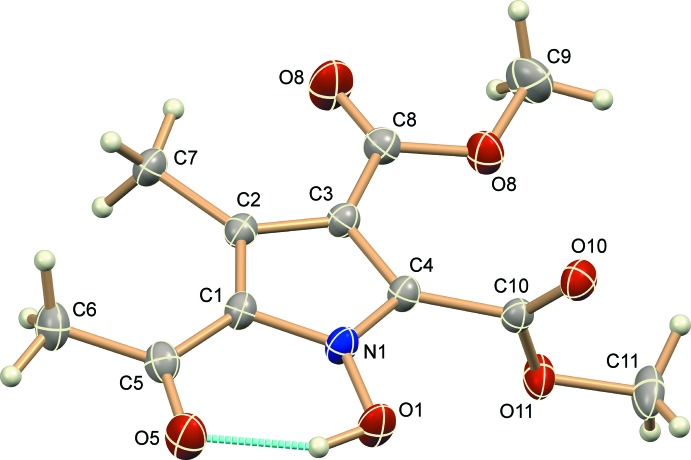
Molecular structure of the title compound with non-hydrogen atoms labeled. Displacement ellipsoids are shown at the 60% probability level.

## supplementary crystallographic information

### 1. Comment

The title compound was synthesized by a published procedure (Hekmatshoar *et al.*, 2008) with the goal of preparing the corresponding nitroxide by oxidation. The molecular structure is shown in Fig. 1. The compound proved resistant to several oxidizing conditions commonly used to convert *N*-hydroxylamines to nitroxides. Resistance of the title compound to oxidation may be attributable to the relatively strong hydrogen bond formed between the *N*-hydroxyl hydrogen and the carbonyl oxygen of the acetyl group in the 5-position. Modulation of redox properties by hydrogen bonding has been documented for quinones (see Guin *et al.* (2011) for review, Gupta and Linschitz (1997) and Feldman *et al.* (2007) for studies on specific series of benzoquinones and naphthoquinones, respectively). Existence of the intramolecular H-bond in the title compound is unsurprising, since in organic crystals where intramolecular hydrogen bonding would result in a planar 6-membered ring structure, the H-bond is almost always observed (Bilton *et al.*, 2000). The observed O–H···O donor-acceptor distance (2.558 Å) is significantly shorter than the mean of 2.692 Å found for 8493 organic crystal structures in the Cambridge Structural Database in which the H-bond closes a 6-membered ring (Galek *et al.*, 2010). Likewise, the observed O–H···O bond angle (150.8°) is significantly greater than the mean of 137.8° found for the same set of 8493 structures (Galek *et al.*, 2010). These comparisons suggest that the intramolecular H-bond in the title compound is stronger than average. Finally, it may be interesting to note that, in the original paper reporting the synthesis of the title compound (Hekmatshoar *et al.*, 2008), the authors suggested H-bonding between the *N*-hydroxyl hydrogen and the carbonyl oxygen of the ester group in the 2-position of the pyrrole ring.

### 2. Experimental

The title compound was prepared by the procedure of Hekmatshoar *et al.* (2008) in an effort to synthesize the corresponding nitroxide. The compound was subjected to four oxidation reactions: 1) *m*-chloroperbenzoic acid in CH_2_Cl_2_, 2) hydrogen peroxide–sodium tungstate in methanol/acetonitrile, 3) nickel peroxide in benzene, and 4) lead dioxide in benzene (Keana *et al.*, 1988). In each case, no nitroxide was isolated, and only the title compound was recovered.

### 3. Refinement

Positions of all H atoms were calculated from geometric considerations. H atoms were refined as riding on the attached C atoms. Orientation of CH_3_ groups was optimized. For all H atoms, *U*_iso_ was refined but constrained to be equal within CH_3_ groups.

### Crystal data

**Table:**

C_11_H_13_NO_6_	*F*(000) = 536
*M**_r_* = 255.22	*D*_x_ = 1.439 Mg m^−^^3^
Monoclinic, *P*2_1_/*c*	Mo *K*α radiation, λ = 0.71073 Å
Hall symbol: -P 2ybc	Cell parameters from 13127 reflections
*a* = 10.3893 (8) Å	θ = 2.4–31.0°
*b* = 15.1803 (12) Å	µ = 0.12 mm^−^^1^
*c* = 7.5789 (6) Å	*T* = 150 K
β = 99.630 (1)°	Prism, colourless
*V* = 1178.45 (16) Å^3^	0.52 × 0.43 × 0.31 mm
*Z* = 4	

### Data collection

**Table:**

Bruker SMART APEXII diffractometer	3437 independent reflections
Radiation source: fine-focus sealed tube	2836 reflections with *I* > 2σ(*I*)
Graphite monochromator	*R*_int_ = 0.014
Detector resolution: 8.333 pixels mm^-1^	θ_max_ = 30.0°, θ_min_ = 2.0°
φ and ω scans	*h* = −14→14
Absorption correction: multi-scan (*SADABS*; Sheldrick, 1996)	*k* = −21→21
*T*_min_ = 0.888, *T*_max_ = 0.964	*l* = −10→10
19259 measured reflections	

### Refinement

**Table:**

Refinement on *F*^2^	Primary atom site location: structure-invariant direct methods
Least-squares matrix: full	Secondary atom site location: difference Fourier map
*R*[*F*^2^ > 2σ(*F*^2^)] = 0.031	Hydrogen site location: difference Fourier map
*wR*(*F*^2^) = 0.064	All H-atom parameters refined
*S* = 1.00	*w* = 1/[σ^2^(*F*_o_^2^) + (0.01*P*)^2^ + 0.4962*P*], *P* = (max(*F*_o_^2^,0) + 2*F*_c_^2^)/3
3437 reflections	(Δ/σ)_max_ < 0.001
215 parameters	Δρ_max_ = 0.35 e Å^−^^3^
0 restraints	Δρ_min_ = −0.19 e Å^−^^3^

### Special details

**Table:**

Geometry. All e.s.d.'s (except the e.s.d. in the dihedral angle between two l.s. planes) are estimated using the full covariance matrix. The cell e.s.d.'s are taken into account individually in the estimation of e.s.d.'s in distances, angles and torsion angles; correlations between e.s.d.'s in cell parameters are only used when they are defined by crystal symmetry. An approximate (isotropic) treatment of cell e.s.d.'s is used for estimating e.s.d.'s involving l.s. planes.
Refinement. Refinement of *F*^2^ against ALL reflections. The weighted *R*-factor *wR* and goodness of fit *S* are based on *F*^2^, conventional *R*-factors *R* are based on *F*, with *F* set to zero for negative *F*^2^. The threshold expression of *F*^2^ > σ(*F*^2^) is used only for calculating *R*-factors(gt) *etc*. and is not relevant to the choice of reflections for refinement. *R*-factors based on *F*^2^ are statistically about twice as large as those based on *F*, and *R*-factors based on ALL data will be even larger. For all H atoms both coordinate and isotropic displacement parameters were freely refined.All H atoms were located from the difference Fourier maps and refined unconstrained including isotropic displacement parameters.

### Fractional atomic coordinates and isotropic or equivalent isotropic displacement parameters (Å^2^)

**Table:**

	*x*	*y*	*z*	*U*_iso_*/*U*_eq_	
N1	0.58967 (7)	0.15991 (5)	0.01532 (11)	0.02245 (15)	
C1	0.57608 (9)	0.25121 (6)	0.00825 (12)	0.02170 (17)	
C2	0.69238 (9)	0.28546 (6)	0.10473 (12)	0.02101 (16)	
C3	0.77256 (8)	0.21282 (6)	0.16540 (12)	0.02102 (16)	
C4	0.70478 (8)	0.13542 (6)	0.10655 (12)	0.02093 (17)	
O1	0.49892 (7)	0.09969 (5)	−0.06513 (11)	0.03059 (16)	
H1	0.4367 (15)	0.1346 (10)	−0.121 (2)	0.055 (4)*	
C5	0.45803 (9)	0.28874 (6)	−0.09308 (13)	0.02522 (18)	
O5	0.37225 (7)	0.23953 (5)	−0.17286 (11)	0.03488 (18)	
C6	0.44010 (11)	0.38675 (7)	−0.10178 (16)	0.0318 (2)	
H61	0.3632 (15)	0.3989 (10)	−0.190 (2)	0.056 (4)*	
H62	0.4261 (14)	0.4098 (10)	0.012 (2)	0.048 (4)*	
H63	0.5176 (13)	0.4162 (9)	−0.1332 (17)	0.039 (4)*	
C7	0.72552 (11)	0.38090 (6)	0.13774 (14)	0.02725 (19)	
H71	0.8031 (14)	0.3861 (10)	0.227 (2)	0.050 (4)*	
H72	0.6559 (14)	0.4127 (9)	0.1794 (18)	0.044 (4)*	
H73	0.7376 (13)	0.4103 (9)	0.0300 (19)	0.044 (4)*	
C8	0.90325 (9)	0.21540 (6)	0.27407 (13)	0.02425 (18)	
O8	0.96516 (8)	0.28077 (5)	0.32002 (13)	0.0425 (2)	
O9	0.94825 (7)	0.13382 (5)	0.31885 (10)	0.02999 (16)	
C9	1.07658 (11)	0.13084 (9)	0.42886 (18)	0.0394 (3)	
H91	1.0973 (16)	0.0679 (11)	0.439 (2)	0.062 (5)*	
H92	1.1394 (14)	0.1618 (10)	0.3656 (19)	0.046 (4)*	
H93	1.0726 (13)	0.1588 (10)	0.543 (2)	0.046 (4)*	
C10	0.74086 (8)	0.04013 (6)	0.13628 (12)	0.02129 (17)	
O10	0.70851 (7)	−0.00387 (4)	0.25304 (10)	0.02722 (15)	
O11	0.81287 (7)	0.01306 (5)	0.01819 (10)	0.02943 (16)	
C11	0.86684 (14)	−0.07523 (8)	0.04835 (18)	0.0383 (3)	
H111	0.9238 (14)	−0.0816 (9)	−0.0377 (19)	0.046 (4)*	
H112	0.9172 (15)	−0.0775 (11)	0.170 (2)	0.060 (5)*	
H113	0.7977 (15)	−0.1177 (11)	0.037 (2)	0.059 (5)*	

### Atomic displacement parameters (Å^2^)

**Table:**

	*U*^11^	*U*^22^	*U*^33^	*U*^12^	*U*^13^	*U*^23^
N1	0.0195 (3)	0.0176 (3)	0.0295 (4)	−0.0019 (3)	0.0019 (3)	−0.0014 (3)
C1	0.0216 (4)	0.0181 (4)	0.0260 (4)	0.0008 (3)	0.0056 (3)	0.0002 (3)
C2	0.0233 (4)	0.0185 (4)	0.0220 (4)	−0.0006 (3)	0.0061 (3)	−0.0007 (3)
C3	0.0203 (4)	0.0196 (4)	0.0232 (4)	−0.0016 (3)	0.0038 (3)	−0.0009 (3)
C4	0.0192 (4)	0.0186 (4)	0.0250 (4)	−0.0002 (3)	0.0040 (3)	−0.0004 (3)
O1	0.0225 (3)	0.0219 (3)	0.0438 (4)	−0.0053 (3)	−0.0046 (3)	−0.0031 (3)
C5	0.0224 (4)	0.0257 (4)	0.0284 (4)	0.0036 (3)	0.0068 (3)	0.0028 (4)
O5	0.0246 (3)	0.0322 (4)	0.0446 (4)	0.0018 (3)	−0.0036 (3)	0.0005 (3)
C6	0.0282 (5)	0.0256 (5)	0.0414 (6)	0.0066 (4)	0.0055 (4)	0.0056 (4)
C7	0.0338 (5)	0.0184 (4)	0.0291 (5)	−0.0026 (4)	0.0039 (4)	−0.0019 (4)
C8	0.0223 (4)	0.0251 (4)	0.0253 (4)	−0.0020 (3)	0.0037 (3)	0.0002 (3)
O8	0.0323 (4)	0.0298 (4)	0.0591 (5)	−0.0082 (3)	−0.0103 (4)	−0.0025 (4)
O9	0.0226 (3)	0.0286 (4)	0.0362 (4)	0.0011 (3)	−0.0027 (3)	0.0030 (3)
C9	0.0268 (5)	0.0473 (7)	0.0399 (6)	0.0063 (5)	−0.0063 (4)	0.0017 (5)
C10	0.0193 (4)	0.0186 (4)	0.0247 (4)	−0.0003 (3)	0.0001 (3)	−0.0014 (3)
O10	0.0305 (3)	0.0218 (3)	0.0303 (3)	−0.0020 (3)	0.0079 (3)	0.0013 (3)
O11	0.0368 (4)	0.0229 (3)	0.0308 (4)	0.0106 (3)	0.0120 (3)	0.0057 (3)
C11	0.0488 (7)	0.0259 (5)	0.0442 (6)	0.0169 (5)	0.0190 (6)	0.0075 (5)

### Geometric parameters (Å, º)

**Table:**

N1—C4	1.3304 (11)	C7—H71	0.963 (15)
N1—O1	1.3799 (10)	C7—H72	0.965 (14)
N1—C1	1.3932 (11)	C7—H73	0.957 (14)
C1—C2	1.4033 (12)	C8—O8	1.2020 (12)
C1—C5	1.4503 (13)	C8—O9	1.3470 (12)
C2—C3	1.4118 (12)	O9—C9	1.4501 (13)
C2—C7	1.5005 (13)	C9—H91	0.980 (17)
C3—C4	1.4037 (12)	C9—H92	0.991 (15)
C3—C8	1.4661 (12)	C9—H93	0.971 (15)
C4—C10	1.5018 (12)	C10—O10	1.2007 (11)
O1—H1	0.888 (16)	C10—O11	1.3242 (11)
C5—O5	1.2399 (12)	O11—C11	1.4561 (12)
C5—C6	1.4995 (14)	C11—H111	0.956 (14)
C6—H61	0.968 (15)	C11—H112	0.981 (16)
C6—H62	0.963 (15)	C11—H113	0.958 (16)
C6—H63	0.985 (14)		
			
C4—N1—O1	122.20 (8)	C2—C7—H72	112.0 (8)
C4—N1—C1	111.99 (7)	H71—C7—H72	108.4 (11)
O1—N1—C1	125.78 (8)	C2—C7—H73	111.6 (8)
N1—C1—C2	106.00 (8)	H71—C7—H73	110.1 (12)
N1—C1—C5	118.84 (8)	H72—C7—H73	104.9 (11)
C2—C1—C5	135.11 (8)	O8—C8—O9	122.66 (9)
C1—C2—C3	106.86 (8)	O8—C8—C3	125.82 (9)
C1—C2—C7	126.74 (8)	O9—C8—C3	111.52 (8)
C3—C2—C7	126.40 (8)	C8—O9—C9	114.87 (8)
C4—C3—C2	108.24 (8)	O9—C9—H91	104.2 (10)
C4—C3—C8	124.69 (8)	O9—C9—H92	108.9 (8)
C2—C3—C8	127.06 (8)	H91—C9—H92	110.4 (12)
N1—C4—C3	106.90 (8)	O9—C9—H93	109.0 (8)
N1—C4—C10	121.82 (8)	H91—C9—H93	113.3 (13)
C3—C4—C10	131.24 (8)	H92—C9—H93	110.7 (12)
N1—O1—H1	101.8 (10)	O10—C10—O11	125.81 (8)
O5—C5—C1	119.78 (9)	O10—C10—C4	123.55 (8)
O5—C5—C6	120.16 (9)	O11—C10—C4	110.64 (8)
C1—C5—C6	120.06 (9)	C10—O11—C11	115.20 (8)
C5—C6—H61	107.5 (9)	O11—C11—H111	104.6 (9)
C5—C6—H62	111.0 (9)	O11—C11—H112	108.1 (10)
H61—C6—H62	108.4 (12)	H111—C11—H112	110.1 (12)
C5—C6—H63	111.2 (8)	O11—C11—H113	110.0 (9)
H61—C6—H63	111.1 (11)	H111—C11—H113	114.3 (13)
H62—C6—H63	107.6 (11)	H112—C11—H113	109.5 (13)
C2—C7—H71	109.7 (9)		
			
C4—N1—C1—C2	−0.55 (11)	C2—C3—C4—C10	−177.93 (9)
O1—N1—C1—C2	−178.69 (8)	C8—C3—C4—C10	1.01 (15)
C4—N1—C1—C5	177.39 (8)	N1—C1—C5—O5	−0.77 (14)
O1—N1—C1—C5	−0.75 (14)	C2—C1—C5—O5	176.43 (10)
N1—C1—C2—C3	0.48 (10)	N1—C1—C5—C6	179.45 (9)
C5—C1—C2—C3	−176.97 (10)	C2—C1—C5—C6	−3.36 (16)
N1—C1—C2—C7	−179.43 (9)	C4—C3—C8—O8	176.89 (10)
C5—C1—C2—C7	3.13 (17)	C2—C3—C8—O8	−4.37 (16)
C1—C2—C3—C4	−0.26 (10)	C4—C3—C8—O9	−3.19 (13)
C7—C2—C3—C4	179.64 (9)	C2—C3—C8—O9	175.56 (9)
C1—C2—C3—C8	−179.17 (9)	O8—C8—O9—C9	0.77 (15)
C7—C2—C3—C8	0.73 (15)	C3—C8—O9—C9	−179.15 (9)
O1—N1—C4—C3	178.61 (8)	N1—C4—C10—O10	−82.60 (12)
C1—N1—C4—C3	0.39 (11)	C3—C4—C10—O10	94.99 (12)
O1—N1—C4—C10	−3.29 (14)	N1—C4—C10—O11	97.95 (10)
C1—N1—C4—C10	178.50 (8)	C3—C4—C10—O11	−84.45 (12)
C2—C3—C4—N1	−0.07 (10)	O10—C10—O11—C11	−6.69 (15)
C8—C3—C4—N1	178.87 (8)	C4—C10—O11—C11	172.74 (9)

### Hydrogen-bond geometry (Å, º)

**Table:**

*D*—H···*A*	*D*—H	H···*A*	*D*···*A*	*D*—H···*A*
O1—H1···O5	0.888 (16)	1.746 (16)	2.5583 (11)	150.8 (14)

## References

[bb1] Bilton, C., Allen, F. H., Shields, G. P. & Howard, J. A. K. (2000). *Acta Cryst.* B**56**, 849–856. 10.1107/S0108768100003694 11006561

[bb2] Bruker (2010). *APEX2*, *SAINT* and *XSHELL* Bruker AXS Inc., Madison, Wisconsin, USA.

[bb3] Feldman, K. S., Hester, D. K. II & Golbeck, J. H. (2007). *Bioorg. Med. Chem. Lett.* **17**, 4891–4894. 10.1016/j.bmcl.2007.06.041 PMC2084345 17596943

[bb4] Galek, P. T. A., Fábián, L. & Allen, F. H. (2010). *Acta Cryst.* B**66**, 237–252. 10.1107/S0108768110003988 20305358

[bb5] Guin, P. S., Das, S. & Mandal, P. C. (2011). *Int. J. Electrochem.* vol. 2011, Article ID 816202, 22 pages, 10.4061/2011/816202.

[bb6] Gupta, N. & Linschitz, H. (1997). *J. Am. Chem. Soc.* **119**, 6384–6391.

[bb7] Hekmatshoar, R., Nouri, R. & Beheshtiha, S. Y. Sh. (2008). *Heteroat. Chem.* **19**, 100–103.

[bb8] Keana, J. F. W., Heo, G. S., Mann, J. S., Van Nice, F. L., Lex, L., Prabhu, V. S. & Ferguson, G. (1988). *J. Org. Chem.* **53**, 2268–2274.

[bb9] Macrae, C. F., Bruno, I. J., Chisholm, J. A., Edgington, P. R., McCabe, P., Pidcock, E., Rodriguez-Monge, L., Taylor, R., van de Streek, J. & Wood, P. A. (2008). *J. Appl. Cryst.* **41**, 466–470.

[bb10] Sheldrick, G. M. (1996). *SADABS* University of Göttingen, Germany.

[bb11] Sheldrick, G. M. (2008). *Acta Cryst.* A**64**, 112–122. 10.1107/S0108767307043930 18156677

[bb12] Westrip, S. P. (2010). *J. Appl. Cryst.* **43**, 920–925.

